# Psychological wellbeing, family cohesion, and purposeful life in male prisoners: A cross-sectional study

**DOI:** 10.3389/fpsyt.2022.1054149

**Published:** 2023-01-05

**Authors:** Hooshang Ghazanfari, Sakineh Miri, Mozhgan Taebi, Jamileh Farokhzadian

**Affiliations:** ^1^Department of Community Health Nursing, Razi Faculty of Nursing and Midwifery, Kerman University of Medical Sciences, Kerman, Iran; ^2^Department of Psychiatric Nursing, Razi Faculty of Nursing and Midwifery, Kerman University of Medical Sciences, Kerman, Iran; ^3^Department of Anesthesia, Faculty of Allied Medicine, Kerman University of Medical Sciences, Kerman, Iran; ^4^Nursing Research Center, Kerman University of Medical Sciences, Kerman, Iran

**Keywords:** inmates, imprisoned men, mental wellbeing, prisoners, family cohesion, meaning in life

## Abstract

**Background:**

Psychological wellbeing, family cohesion, and purposeful life are important determinants of the prisoners’ overall wellbeing and health; therefore, their evaluation is extremely important in prisoners as a vulnerable group.

**Objective:**

This study evaluated psychological wellbeing, family cohesion, purposeful life, and their correlations in male prisoners.

**Methods:**

This cross-sectional study used simple random sampling to select 259 male prisoners. Data were collected using questionnaires of Ryff psychological wellbeing, Fischer family cohesion, and Crumbaugh and Maholick purpose in life.

**Results:**

Majority of (78%) the participants were 20–40 years old and married (59%). The mean scores of psychological wellbeing, family cohesion, and purposeful life of the male prisoners were moderate. Psychological wellbeing was directly correlated to family cohesion in male prisoners, but it had no significant correlation with a purposeful life. Family cohesion was not significantly correlated to a purposeful life.

**Conclusion:**

Regarding the moderate level of psychological wellbeing in prisoners, it is suggested to pay more attention to educational and supportive programs in prisons for promoting such indicators in prisoners.

## Introduction

More than 10 million prisoners are available worldwide, and approximately 30 million prisoners are released annually (1). More than 7 million prisoners are living in low-income and middle-income countries (LMICs), comprising about 70% of the world’s total prison population (2). Many of whom are imprisoned due to drug-related crimes. About 225,000 Iranian prisoners are above 18 years old, with 3.91% of them being female (3), while under-18 years-old prisoners are kept in the juvenile correctional and rehabilitation center (4).

The number of prisoners differs from one country to another, and it is difficult to compare the number of criminals in different societies because police and judicial institutions in every country have different rules for imprisoning criminals (5), and their rules may change with the passage of time (6). The number of male prisoners is much higher than that of females all over the world (eight male prisoners vs. two female prisoners) (7). Moreover, the pathways to prison differ between male and female (8, 9), and socio-psychological problems, HIV prevalence, and related risk behaviors, such as drug injection among male prisoners is higher than that in female prisoners. Therefore, it is important to study male prisoners (10).

Prisoners are vulnerable to multiple problems, with many restrictions and deprivations in prison adding to these problems (11). They suffer from poor relationships and social rejection because of the norms and subcultures in prison and separation from the family. Such problems decrease their personal and social adjustment, as well as their mental health and safety, but some prisoners are efficient and young and must return to social environment after their release (12). Prison is a potentially important stressor in either the formation or exacerbation of prisoners’ psychological problems, such as low level of psychological wellbeing (PWB) (13).

Psychological wellbeing is among the most important determinant of global wellbeing and health of prisoners. The PWB is defined as an attempt to realize one’s potential capabilities and includes six characteristics of “positive relationships with others,” “self-acceptance,” “personal growth,” “purpose in life,” “environmental mastery,” and “autonomy,” which indicate one’s mental balance and health (14). Wellbeing generally predicts a variety of vital outcomes, including a sense of life satisfaction, and greater success in job and relationships (15). Studies have shown prisoners’ low level of PWB, so its assessment in prisoners is important because they are vulnerable (16, 17).

Poor family cohesion contributes to one’s diversion and criminal behavior, delinquency and consequently imprisonment. Upon entering prison, this problem has destructive effects on family relationships (18). Family cohesion (FC), a determining factor for PWB (19), is defined as the emotional bond between family members. FC is associated with adolescents’ internal and external problems and predicts their future behaviors. External psychological symptoms, such as academic failure, isolation, violence, and hyperactivity, and internal symptoms, such as anxiety and depression, may also transmit to adulthood (20).

Imprisonment seriously disrupts planning and purpose in life (PL) (21). PL is a positive structure that can better predict and promote well-being (22). PL is an important religious, philosophical and psychological topic and has various definitions in different fields; however, it is usually defined as one’s sense of function and meaning in life. “Purpose in life” and “meaning in life” are often used interchangeably (22).

A review of the literature showed that PL was an important and positive personality trait and predicted health outcomes (23) as well as a higher level of PWB in present and future (24). Goodman et al. (25) showed a significant positive relationship between family attachment and PL in men and women. Alhussain et al. (26) reported a significant relationship between PWB and FC in young people (26). Another study on students showed that the higher the FC, the higher the PWB (27).

Problems of prisoners are usually ignored and neglected by community health nurses and social workers, who can render professional services to prisoners and their families by assessing the PWB, FC, PL, and their inter-relationships. They also can design social work interventions and educational programs to increase the PWB, FC, and PL. Given the cultural and contextual nature of these factors, studies are required in different communities to design comprehensive educational interventions. No study has simultaneously examined the three variables of PL, FC, and PWB in prisoners. Most of the studies mentioned above were conducted on non-prisoners and those who had clear differences with prisoners, so the current study aimed to evaluate PWB, FC, PL, and their correlations in male prisoners in Iran.

## Materials and methods

### Design and settings

This cross-sectional study was conducted on male prisoners in a large prison in southeastern Iran.

#### Sampling

The target population of this study included all prisoners aged above 18 years old, who were available at the time of the study (*n* = 4500). According to Cochran’s formula, the sample size was 252 people (α = 0.05, d = 0.06, Z = 1.94). With about a 5% probability of dropout, 265 male prisoners were selected using simple random sampling. Inclusion criteria included prisoners, who could read and write to complete and understand the concepts of the questionnaires, were imprisoned for at least 6 months, had at least one member family, and declared no acute mental and physical illnesses at the time of data collection. The first researcher selected samples using simple random, so he prepared the list of prisoners and recruited the samples using random number table.

### Data collection and instruments

Data were collected using anonymous, self-reported, and paper questionnaires from March to May 2021. To collect data, the first researcher referred to the study setting, distributed the questionnaires among the eligible participants, and explained the study objectives. He also trained prisoners on how to fill out the questionnaires. Finally, 259 prisoners completed the questionnaires and six prisoners did not complete questionnaires (the response rate = 97.73%). four tools were used in the current study.

#### Demographic information questionnaire

It includes age, education, nationality, occupation, number of marriages, length of stay in prison, history of imprisonment, history of drug abuse and its type, history of mental illness, psychiatric drugs, and smoking and type of crime.

#### Ryff psychological wellbeing scale

Ryff developed this self-report scale in 1989. The short 18-item PWBS with six subscales was used in this study: self-acceptance (2, 8, 10), autonomy (9, 12, 18), positive relationships with others (3, 11, 13), purpose in life (5, 14, 16), personal growth (7, 15, 17) and environmental mastery (1, 4, 6). Ryff confirmed the reliability of the original version of PWBS (0.89) using Cronbach’s alpha coefficient, as well as its validity through content validity (28). Researchers confirmed the reliability of the Persian version of this questionnaire (0.81) using Cronbach’s alpha coefficient and experts confirmed its validity (29). The PWBS is scored based on a six-point Likert scale, including strongly agree (6), somewhat agree (5), a little agree (4), a little disagree (3), somewhat disagree (2), and strongly disagree (1). The total score in this questionnaire is between 18 and 108, with scores of 18–48, 49–78, and above 79 considering as poor, moderate, and favorable PWB, respectively.

#### Family standardized cohesion questionnaire

Fischer et al. (30) developed this questionnaire as part of the California Family health Project. The questionnaire has 13 items and four dimensions, including cohesion (1, 5, 6, 10, and 12), cooperation (7, 9, 11, and 13), clarity of rules (2 and 4), and leadership in the family (3 and 8). the questionnaire is scored based on a six-point likert scale ranging from strongly agree (6), somewhat agree (5), a little agree (4), a little disagree (3), somewhat disagree (2), and strongly disagree (1). Fischer et al. reported the internal consistency of the original version to be 0.78 using Cronbach’s alpha (30). Experts confirmed the validity of the Persian version of this questionnaire, and its reliability was above 0.70 using Cronbach’s alpha method (29). In this study, scores of 0–2, 2–4, and 4–6 were considered as poor, moderate, and favorable FC, respectively.

#### Purpose in life questionnaire

Krombugh and Maholick developed this scale in 1969 to measure individual’s sense of purpose or meaning in life. The questionnaire consisted of 20 items and participants chose one answer between one and seven. Scoring, type, and content of the items in this questionnaire are different with the subscale of purpose in life of PWB. In this questionnaire, the minimum and maximum scores are 20 and 140, with scores of 20–60, 60–100, and 100–140 considering as poor, moderate, and high PL, respectively. The reliability coefficient of the original version of PIL was 0.88 using Cronbach’s alpha coefficient. The questionnaire has content validity based on Frankl’s logotherapy theory (31). A study on 250 Iranians reported that the reliability coefficient of the Persian version of the PIL questionnaire was 0.92 using Cronbach alpha. The validity of the Persian version was confirmed by studying the correlation of its scores with life satisfaction, vitality, and positive-negative affection (32).

### Data analysis

The data were analyzed by SPSS 21. First, data normality was determined by the Kolmogorov-Smirnov test. Regarding the normality of quantitative data, the Pearson test, and multivariate linear regression were used. The significance level was considered 0.05.

## Results

### Demographic information

Majority of the participants were 20–40 years old (78%), married (59%), Iranian (96.5%), and had diploma (42.1%). Table 1 shows all demographic information of the male prisoners.

**TABLE 1:** Demographic information of male prisoners (*n* = 259).

Variables	Categories	*n*	%
Age groups	20–40	202	78
	≥41	57	22
Education	Elementary	99	38.2
	Diploma	109	42.1
	Academic	51	19.7
Citizenship	Iranian	250	96.5
	Non-Iranian (Afghans)	9	3.5
Job	Unemployed	5	1.9
	Self-employed	217	83.8
	Employee	18	6.9
	Others	19	7.3
Marital status	Single	75	29
	Married	153	59
	Divorced	28	10.8
	Widower	3	1.2
Number of children in paternal family	1	34	13.2
	2	40	15.4
	3	40	15.4
	≥4	145	56
The number of marriages (time)	1	148	80.03
	2	33	17.9
	≥3	6	1.8
Length of imprisonment	≤6 months	65	25.1
	6 months–2 years	69	26.6
	2–3 years	36	13.9
	>3 years	89	34.4
History of prison (time)	1	117	45.1
	2	83	32
	≥3	59	22.8
History of mental disorders and psychiatric drugs	Yes	92	35.5
	No	167	64.5
History of narcotic substances use (Drug abuse)	Yes	183	70.6
	No	76	29.4
Type of drug abuse	Non-industrial	92	35.5
	Industrial	91	35.1
Smoking	Yes	126	48.6
	No	133	51.4
Type of crime	Substance abuse	109	44
	Substance abuse and theft	6	2.3
	Theft	53	20.3
	Political activity	5	1.9
	Carrying a concealed weapon and theft	1	0.3
	Carrying a concealed weapon and theft	1	0.3
	Not paying back the money one owed	44	16.9
	Contraband	2	0.7
	Not paying back the money one owed, substance abuse	2	0.7
	Fraud	5	1.9
	Not paying a woman’s dowry	4	1.5
	Assault and battery	6	2.3
	Consumption of alcohol	2	0.7
	Forgery	1	0.3
	Selling illegal goods	1	0.3
	Guarantor of a person	2	0.3
	Fence	2	0.3
	Murder	8	3.4
	Rape	1	0.3
	Keeping a disorderly house	1	0.3
	Market disruption	1	0.3
	Kidnapping	1	0.3
	Substance abuse and murder	1	0.3
	Damage to government property	1	0.3

### Description of PWB, FC, and PL

The results showed that the total mean PWB score of the male prisoners was moderate (55.90 ± 11.90), with purpose in life subscale (10.68 ± 3.32) and environmental mastery subscale (8.26 ± 3.39) receiving the highest and lowest mean scores, respectively.

The total mean FC score of male prisoners was moderate (2.67 ± 1.13), with the clarity of rules and cohesion subscales receiving the highest (3.09 ± 1.38) and lowest (2.64 ± 1.18) mean scores, respectively.

The total mean PL score was moderate (79.48 ± 17.32) in male prisoners; the item “I wasted the rest of my life after retirement. I did the most exciting things I ever wanted to do” (4.87 ± 1.07) received the highest mean score, while the item “I am very responsible…. I am very irresponsible” (3.79 ± 2.03) received the lowest mean score (Table 2).

**TABLE 2:** Mean scores of variables and their dimensions in male prisoners.

Variable	Dimensions	Mean ± SD
Psychological wellbeing	Self-acceptance	9.64 ± 3.01
	Autonomy	8.40 ± 3.42
	Positive relationships with others	10.07 ± 3.21
	Purpose in life	10.68 ± 3.32
	Personal growth	8.87 ± 3.40
	Environmental mastery	8.26 ± 3.39
	Total psychological wellbeing	55.95 ± 11.90
Family cohesion	Cohesion	2.64 ± 1.18
	Cooperation	2.52 ± 1.28
	Clarity of rules	3.09 ± 1.38
	Leadership	2.65 ± 1.49
	Total family cohesion	2.67 ± 1.13
Purposeful life	Total purposeful life	79.48 ± 17.32

### Correlation between variables

The results of this study showed a direct correlation between male prisoners’ PWB and FC scores *(r* = 0.46, *p* = 0.001), but PWB had no significant correlation with PL score. In addition, FC score was not significantly correlated with the PL score. All dimensions of PWB had a direct correlation with FC except for positive relationships with others. In addition, all dimensions of FC had direct correlation with PWB. None of the PWB and FC dimensions were related to PL (Table 3).

**TABLE 3:** Correlation between the measured variables.

Variables		Family cohesion					Purposeful life
		Cohesion	Cooperation	Clarity of rules	Leadership	Total family cohesion	
Psychological wellbeing	Self-acceptance	*r* = 0.34 *p* = 0.001*	*r* = 0.28 *p* = 0.001*	*r* = 0.22 *p* = 0.001*	*r* = 0.28 *P* = 0.001*	*r* = 0.35 *P* = 0.001*	*r* = 0.10 *p* = 0.06
	Autonomy	*r* = 0.34 *p* = 0.001*	*r* = 0.27 *p* = 0.001*	*r* = 0.31 *p* = 0.001*	*r* = 0.24 *p* = 0.001*	*r* = 0.35 *P* = 0.001*	*r* = 0.01 *p* = 0.81
	Positive relationships with others	*r* = 0.02 *p* = 0.79	*r* =−0.02 *p* = 0.75	*r* =−0.02 *p* = 0.74	*r* = 0.02 *p* = 0.74	*r* = 0.06 *P* = 0.35	*r* = 0.05 *p* = 0.50
	Purpose in life	*r* = 0.24 *p* = 0.001*	*r* = 0.20 *p* = 0.001*	*r* = 0.23 *p* = 0.001*	*r* = 0.16 *p* = 0.001*	*r* = 0.24 *p* = 0.001*	*r* = 0.02 *p* = 0.64
	Personal growth	*r* = 0.34 *p* = 0.001*	*r* = 0.30 *p* = 0.001*	*r* = 0.23 *p* = 0.001*	*r* = 0.27 *p* = 0.001*	*r* = 0.36 *p* = 0.001*	*r* = 0.02 *p* = 0.71
	Environmental mastery	*r* = 0.24 *p* = 0.001*	*r* = 0.14 *p* = 0.001*	*r* = 0.17 *p* = 0.001*	*r* = 0.15 *p* = 0.001*	*r* = 0.32 *p* = 0.001*	*r* = 0.08 *p* = 0.20
	Total psychological wellbeing	*r* = 0.42 *p* = 0.001*	*r* = 0.35 *p* = 0.001*	*r* = 0.31 *p* = 0.001*	*r* = 0.31 *p* = 0.001*	*r* = 0.46 *P* = 0.001*	*r* = 0.08 *p* = 0.20

Family cohesion	Cohesion	–	–	–	–	–	*r* = 0.02 *p* = 0.75
	Cooperation	–	–	–	–	–	*r* = 0.09 *p* = 0.12
	Clarity of rules	–	–	–	–	–	*r* = 0.08 *p* = 0.19
	Leadership	–	–	–	–	–	*r* = 0.003 *p* = 0.96
	Total family cohesion	–	–	–	–	–	*r* = 0.05 *p* = 0.36

*Significant at level *p* ≤ 0.05.

### Regression analyses

The multivariate linear regression showed that FC (β = 0.27, *p* = 0.009), marital status (β =−0.25, *p* = 0.02), and length of imprisonment (β = 0.36, *p* = 0.002) were the significant predictors of PWB. Therefore, married prisoners with a higher level of FC and imprisoned between 6 months and 2 years had higher scores in PWB (Table 4).

**TABLE 4:** Multivariate regression model for all variables and psychological wellbeing.

Variables	β	*t*	*P*-value
Family cohesion	0.27	2.67	0.009*
Age groups	–0.04	–0.36	0.71
Education	0.03	0.32	0.74
Citizenship	–0.15	–1.28	0.2
Job	0.003	0.03	0.97
Marital status	–0.25	–2.24	0.02*
The number of marriages (time)	0.05	0.54	0.58
Length of imprisonment	0.36	3.29	0.002*
History of prison (time)	0.06	–0.61	0.54
Number of children in paternal family	–0.06	–0.61	0.54
History of mental disorders and psychiatric drugs	–0.11	–0.97	0.33
History of narcotic substances use (Drug abuse)	–0.03	–0.31	0.75
Type of drug abuse	0.02	0.25	0.8
Smoking	0.01	0.09	0.92
Type of crime	–0.14	–1.36	0.17

*Significant at level *p* ≤ 0.05.

## Discussion

The current study showed moderate PWB, FC, and PL, as well as a direct correlation between PWB and FC in male prisoners. One study on young prisoners in Iran supported our results and reported moderate PWB of the male prisoners (33). Modarres et al. (34) indicated that counseling and training were effective in promoting mental health indicators among prisoners (34). They believed that prisons were not closed and passive environments and do not merely seek to punish individuals; rather, they considered other aspects of their lives. Such results may be due to the organizational goals of prisons, including therapeutic, psychiatric, social work, rehabilitation, and correctional activities in prisons. Therefore, relevant experts perform a wide range of activities, such as cultural, religious, artistic, scientific, and vocational programs, which can be effective in improving the PWB of prisoners. In addition, the factors and conditions that prisoners deal with before imprisonment may affect their PWB. As PWB develops over time, it can be concluded that prisoners have suffered psychologically before their imprisonment. Moreover, depending on the psychological and personality characteristics of prisoners, they might have had various exciting experiences in their past lives.

Purpose in life, the PWB subscale, received the highest mean score, which may be due to the type and content of the items in this subscale. For example, “Sometimes I feel I have done everything in my life” indicates that the prisoners are constantly worried about employment, family, financial status, and other important issues of post-release life, so they have the opportunity to think about their lives as well as learn the psychological training provided in prison. Environmental mastery received the lowest score, which was expectable because of prisoners’ conditions and limitations. Prisoners are in a limited environment, have no choice in sleeping, waking up, exercising, training, or other programs, and must perform all their daily activities collectively and systematically.

The results revealed moderate FC of the male prisoners; social work and cultural activities in prison may have contributed to improving outcomes. The social work sector in prison attempts to improve conditions by strengthening prisoners’ family relationships and interests through phone calls, in-person visits, and temporary release from prison. Datchi et al. (37) confirmed the role of family support and family -centered programs in bringing prisoners back to society with favorable wellbeing and family conditions (35). Hall et al. (36) concluded that identifying prisoners’ skills and strengths strengthened their cooperation and involvement with their family members (36), but a study on family members of individuals with substance use disorder reported a poor level of FC (37).

Our results showed that the male prisoners’ PL was moderate. Various factors may affect prisoners’ PL, such as socially adverse conditions, poverty, mental health problems, and lack of awareness of the PL skills before imprisonment and disruptions in the process of living and planning due to imprisonment. When people are imprisoned, their planning and PL are disrupted.

The highest mean score was related to item “I wasted the rest of my life after retirement. I did the most exciting things I ever wanted to do”. This result can be explained by prisoners’ environmental and psychological conditions. Due to the physical and social constraints during their imprisonment, prisoners feel sad about exciting recreation that cannot be done in prison. Therefore, doing something exciting has become a wish for someone who has been in prison for several years. The lowest score was related to item “I am a very responsible person. I am an irresponsible person.” Prison limitations made prisoners not feel good about responsibility, so they did not get good scores.

The results of this study showed that PWB scores of male prisoners were significantly and directly correlated with their FC scores and FC was a predictor of PWB. These results are consistent with those of other studies (26, 38, 39). One reason for this similarity may be related to the important role of the family in different aspects of life, including mental health. Boyraz and Sayger (40) emphasized that increasing parental FC and wellbeing might contribute to the health and wellbeing of children (40). Farajzadegan et al. (38) found that family functioning had a direct and indirect impact on quality of life and wellbeing and that family was important in improving wellbeing (38).

Our results showed no significant correlation between PWB and PL in male prisoners. These results were not consistent with previous studies (24, 41), indicating that PWB was significantly correlated with PL score. One reason why these studies are inconsistent with the present study may be that the studies were conducted on normal groups, while prisoners are vulnerable with special circumstances.

The results of this study showed that the FC score in male prisoners had no significant correlation with their PL score. This result was not consistent with two studies (25, 42), which reported an important role of the family function in identifying and finding meaning in life. Another study emphasized the impact of family on PL with the mediating role of sense of belonging (25). The reasons for this discrepancy may include the type of training and social work provided in prison, as well as the conditions of prisoners. In addition, several factors may affect the correlation between these variables, which have not been examined in this study. For example, PL is based on the time and environment of the individual, and prisoners’ environment and decision making are limited.

## Limitations

The study population was male prisoners in a prison in southeastern Iran. Therefore, generalization of the results to other communities should be performed with caution. It is suggested that longitudinal studies be conducted to provide more detailed information about the changes in the studied variables from prisoners’ imprisonment to their release in different cultures and contexts.

## Conclusion

The results showed that male prisoners’ PWB, FC, and PL were moderate, and the prisoners’ PWB had a direct correlation with their FC. The results highlighted some important practical implications for the healthcare providers, in particular, community health nurses and social workers. They are recommended to design specific interventions, such as rehabilitative, supportive, cultural, and educational programs for prisoners. Researchers should explore the effectiveness of these interventions to improve prisoners’ PWB, FC, and PL and determine factors affecting these variables, as well as the investigative needs of vulnerable groups, such as prisoners.

## Data availability statement

The raw data supporting the conclusions of this article will be made available by the authors, without undue reservation.

## Ethics statement

The studies involving human participants were reviewed and approved by this manuscript, derived from a master’s thesis in nursing (project No. 97000602), was approved by the Ethics Committee of Kerman University of Medical Sciences (Ethics code of IR.KMU.REC.1397.524). At the request of the Ethics Committee, the present study was conducted in accordance with the Declaration of Helsinki and Ethics Publication on Committee (COPE). The researcher presented the letter of introduction to collect data and received permission from the administrative division of prison. He explained the research and its goals to the participants and obtained their consent. The confidentiality of information was respected and the results of the research were reported to the relevant setting. The patients/participants provided their written informed consent to participate in this study.

## Author contributions

All authors involved in the design and preparation of this research, equally contributed to writing the manuscript, and reviewed and approved the final manuscript.
